# Enhancing probiotic stability in industrial processes

**DOI:** 10.3402/mehd.v23i0.18562

**Published:** 2012-06-18

**Authors:** Miguel Gueimonde, Borja Sánchez

**Affiliations:** Department of Microbiology and Biochemistry of Dairy Products, Instituto de Productos Lácteos de Asturias (IPLA-CSIC), Villaviciosa, Asturias, Spain

**Keywords:** probiotics, Lactobacillus, Bifidobacterium, stability, viability

## Abstract

**Background:**

Manufacture of probiotic products involves industrial processes that reduce the viability of the strains. This lost of viability constitutes an economic burden for manufacturers, compromising the efficacy of the product and preventing the inclusion of probiotics in many product categories. Different strategies have been used to improve probiotic stability during industrial processes. These include technological approaches, such as the modification of production parameters or the reformulation of products, as well as microbiological approaches focused on the strain intrinsic resistance. Among the later, both selection of natural strains with the desired properties and stress-adaptation of strains have been widely used.

**Conclusion:**

During recent years, the knowledge acquired on the molecular basis of stress-tolerance of probiotics has increased our understanding on their responses to industrial stresses. This knowledge on stress-response may nowadays be used for the selection of the best strains and industrial conditions in terms of probiotic stability in the final product.

During the last years, there has been an increasing commercial interest in the inclusion of probiotic strains in different products. However, most of the currently used probiotics are fastidious microorganisms, nutritionally demanding and very sensitive to environmental conditions, and often the product manufacture and storage involves processes that reduce the viability of the strains. This constitutes an economic burden for manufacturers and compromises the efficacy of the probiotic product, limiting the inclusion of probiotics in many product categories. In addition, stability, not only in terms of viability but also in terms of metabolic and functional activity, is needed to maintain the desired sensorial attributes and to provide the claimed health benefit during the whole shelf-life of the product. The application of different strategies to enhance probiotic stability and functionality has been the subject of several recent reviews (1–3).

Many different conditions present during the manufacture and storage of the product may affect the stability of probiotics; these include, among others, temperature, pH, water activity (a_w_), oxygen content or the presence of chemicals, and other microorganisms (1). These factors, starting from the strain production process to the storage conditions of the final product, may have a profound effect on the stability and properties of probiotics. Moreover, stability in the product might not be enough, and it is also important that, following consumption, probiotics have to remain viable at sufficient levels during the gastrointestinal tract (GIT) transit.

Industrial processes may be modified to enhance the stability of the strains. To this regard, the selection of the best suited culture medium composition and cell-protectants may positively influence strain survival (4, 5). The chemical composition of the product may also play a role in stability, the presence of antimicrobial compounds and certain food additives being detrimental. Most probiotic strains are obligate anaerobes or facultative anaerobic microorganisms and, therefore, oxygen content is also relevant. In this case, the solutions adopted have been either to reduce oxygen permeation into the food or introducing oxygen scavengers for reducing its redox potential (6). Cells can also be physically protected by means of microencapsulation, which has been reported to improve stability of strains and to confer tolerance to GIT conditions (7).

## Enhancing stress tolerance of probiotic strains

The intrinsic stress-tolerance of the strains seems to be a critical factor in the overall resistance to manufacture and storage of probiotic products. Therefore, apart to the different technological solutions, enhancing the strain intrinsic tolerance is of great industrial interest. Different approaches can be used to this end (3), which can be clustered into three main categories: selection of naturally occurring strains, stress adaptation of naturally occurring strains, and genetic modification of the strains. The first two approaches use already existing diversity and genetic potential, whilst the last one would imply genetic manipulation leading to a genetically modified organism (GMO).

### Selection of the best suited naturally occurring strains

Different strains show large differences in their ability to cope with different manufacturing and storage conditions. Therefore, the initial screening and selection of those naturally occurring strains showing better properties constitutes a primary target for enhancing stability in probiotic products. Among the different probiotic products, yoghurts and fermented milks are the best established in the market. Sensitivity of probiotics to the typical environmental factors present in fermented milks (such as oxygen and acidic pH) is variable. A frequent phenomenon that affects the stability of probiotics in these products is the so-called *postacidification* or production of acid by the starter strains during refrigerated storage. An option to minimize this phenomenon is the selection of strains lacking the ability to postacidify (8). Another example is the tolerance to oxygen; aerobic conditions are present during the process of manufacture and storage of probiotic products, and thus aerotolerance is a desirable trait for industrial strains. To this regard, the species *Bifidobacterium animalis* subsp. *lactis* is more resistant to stress factors, including oxygen, than other *Bifidobacterium* species being the most frequently used species (9). It has also been suggested that exopolysaccharide (EPS)-producing strains may shown better tolerance to stress (10), and, therefore, EPS-producing strains could be initially selected.

### Adaptation of naturally occurring strains

Probiotic strains can be adapted to better tolerate stressful conditions; however, their adaptation capability is limited by their genomic complement. Lactic acid bacteria have adapted to nutrient-rich environments through evolution. During this process, they have lost many genes, which resulted in small size genomes. This phenomenon, know as *reductive evolution* (11), has limited the genomic potential of these microorganisms. Nevertheless, there is old evidence that selected strains can adapt to stress (12) and strain adaptation has been widely applied. Three main approaches have been used to this end: stress pretreatments, mutagenesis, and selective pressure. Of these, the last two involve changes in the genetic content of the strain, whilst the first one is limited to physiological changes.

#### Stress pretreatments

It has been repeatedly shown that subjecting the strains to sublethal stress before exposing them to the harsh conditions found during elaboration processes influences the stability of the microorganisms during product manufacture and storage. To this regard, acidity and heat are some of the main limiting factors affecting strain survival and it has been shown that preexposure to stress improves the subsequent survival under acidic conditions (13) and after heat-shock (14).

#### Mutagenesis

Random mutagenesis induced by UV light or chemicals has been commonly used in microbiology to obtain strains with altered characteristics or to study different microbial processes. This approach has been successfully used in probiotics research for, among others, increasing the stability of *B. animalis* ssp. *lactis* in low pH products (15). This approach can also be used to improve the stability of the product in terms of sensorial attributes, for instance, metabolic activity of bifidobacteria during manufacture or storage of food is often not desirable, since the production of large amounts of acetic acid may result in undesirable flavors. However, the lack of metabolic activity would compromise bifidobacterial stability. Recently, new strains of bifidobacteria producing low amounts of acetic acid have been obtained by UV mutagenesis; these strains would make possible the elaboration of stable and organoleptically acceptable products based exclusively in bifidobacteria (16).

#### Selective pressure

Resistant derivatives may be obtained by exposing sensitive strains to a selective pressure (stress factor). Very often, these derivatives present a stable phenotype and cross-resistance to other stresses, which is advantageous in terms of stability in industrial processes. This approach has been used, in both lactobacilli and bifidobacteria, to obtain derivative strains with improved heat (17, du Toit et al., unpublished observations), oxygen (18), or acid (19) tolerance. These adapted strains represent a promising option for the development of stable probiotic products. Some recent studies show that the addition of stress-resistant probiotics do not promote remarkable changes in the behavior of starters during manufacture, nor any detrimental effect on the sensory properties of fermented milks (20).

In general, the use of these stress-resistant strains can be useful for improving stability in industrial processes; however, care should be taken as the stress adaptation may alter other properties of the strain (21).

### Genetic modification of the strains

An alternative for increasing stability is the use of genetic engineering. However, especially in Europe, GMOs are not well accepted by consumers. Basically there are two different approaches than can be followed: (1) modify the expression/production of genes already present on the microorganism (homologous expression) and (2) introducing genes from other microbial species (heterologous expression). Examples of both alternatives do exist, for example, overexpression of a chaperone in *Lactobacillus paracasei* was found to increase the strain stability (22), whilst the heterologous expression of the betaine uptake system (BetL) from listeria into *Lactobacillus salivarius* was found to increase tolerance to acid and high osmolar conditions (23).

## Future perspectives

As stated above, different strategies have been used for improving stability of probiotic products. However, very often this has been empirically done, without paying much attention to the mechanisms responsible for stress tolerance. During recent years, with the development of ‘omic’ techniques, the molecular basis of stress-tolerance has been extensively studied (24). Future research in this field must take advantage of the enormous amount of data generated, mainly from proteomic and transcriptomic studies, on stress-response to develop fast and easy methods for the selection of the strains/industrial conditions more suitable in terms of probiotic stability in the final product.

It is also especially important to take into account that the technology applied to improve probiotic stability may modify the functionality of the product. It has been reported that manufacturing processes and matrix may affect the functionality of the strains (25, 26). This underlines the need for stability also on terms of functional properties to ensure that probiotics confer the expected health benefit.
